# Mesopic microperimetry in Stargardt disease: Application and reliability

**DOI:** 10.1111/aos.70072

**Published:** 2026-01-29

**Authors:** Sybren H. Kootstra, Jeroen A. A. H. Pas, Patty P. A. Dhooge, Steffen Schmitz‐Valckenberg, Maurizio Battaglia Parodi, Philipp Herrmann, Frank G. Holz, Andrew J. Lotery, Katarina Stingl, Thomas H. Wheeler‐Schilling, Camiel J. F. Boon, Carel B. Hoyng, Soraprazan Consortium

**Affiliations:** ^1^ Department of Ophthalmology Radboud University Medical Centre Nijmegen GA The Netherlands; ^2^ Donders Institute for Brain, Cognition and Behaviour Radboud University Nijmegen EN The Netherlands; ^3^ Department of Ophthalmology University of Bonn Bonn Germany; ^4^ John A. Moran Eye Centre University of Utah Salt Lake City Utah USA; ^5^ GRADE Reading Centre Bonn Germany; ^6^ Department of Ophthalmology Ospedale San Raffaele Milan Italy; ^7^ Centre for Rare Diseases Bonn (ZSEB) University of Bonn Bonn Germany; ^8^ Faculty of Medicine University of Southampton Southampton UK; ^9^ University Eye Hospital, Centre for Ophthalmology University of Tübingen Tübingen Germany; ^10^ Centre for Rare Eye Diseases University of Tübingen Tübingen Germany; ^11^ Department of Ophthalmology Amsterdam University Medical Centre Amsterdam HV The Netherlands; ^12^ Department of Ophthalmology Leiden University Medical Centre Leiden ZA The Netherlands

**Keywords:** perimetry, retina, stargardt disease, treatment outcome, vision tests

## Abstract

**Purpose:**

Mesopic microperimetry (mMP) is a promising functional endpoint in clinical trials for Stargardt disease type 1 (STGD1). This study evaluated the test–retest variability of mMP and influencing factors, which is essential for ensuring reliability in future STGD1 trials.

**Methods:**

One hundred and fifteen eyes from 68 patients enrolled in the prospective, tertiary, multicentre STArgardt Remofuscin Treatment Trial (STARTT) underwent mMP testing using the macular integrity assessment (MAIA) microperimeter (CenterVue, Padova, Italy) at both the screening (first) and baseline (second) visits of the trial. Test–retest variability was assessed using Bland–Altman analyses and coefficients of repeatability (CoR). Retinal sensitivity metrics included *mean sensitivity* (*MS*) and *pointwise sensitivity* (*PWS*). Other factors including fixation stability, exam duration and learning effect were analysed.

**Results:**

*MS* demonstrated the lowest variability (CoR: 3.53 dB, 95% CI: 3.07–3.99), while *PWS* exhibited the highest (CoR: 12.69 dB, 95% CI: 12.47–12.91). Variability decreased in sensitivity ranges from −1 to 3 dB and 16 to 32 dB and from central to peripheral regions. Test duration (Spearman's *ρ* = 0.609, *p* < 0.001) and fixation losses (Spearman's *ρ* = 0.284, *p* = 0.003) were significantly associated with increased variability. Other fixation stability metrics showed no correlation. No learning effect was observed.

**Conclusions:**

Given its high variability, *PWS* should be used cautiously. *MS* offers lower variability but may mask localised functional changes. A parafoveal ring strategy may improve reliability but requires validation. Limiting test duration to ≤450 seconds and comprehensive operator training are recommended to minimise potential bias.

## 1 INTRODUCTION

With new treatment approaches for Stargardt disease type 1 (STGD1) on the horizon, identifying the most appropriate biomarkers to monitor disease progression is becoming increasingly important.

Several biomarkers are already used to assess disease progression, including best‐corrected visual acuity (BCVA) and outer retinal atrophy measured by fundus autofluorescence (FAF) imaging or spectral domain optical coherence tomography (SD‐OCT) (Andersen et al., 2024; Greenstein et al., 2023; Kong et al., 2018; Lambertus et al., 2016).

BCVA directly reflects visual function, but its decline is not linear, making it a less predictable biomarker of disease progression (Kong et al., 2018; Lambertus et al., 2016; Pas et al., 2024). In contrast, outer retinal atrophy does tend to progress in a relatively linear manner and can be readily measured using FAF and SD‐OCT, although the growth of atrophy does not always reflect changes in visual function (Greenstein et al., 2023; Kong et al., 2018; Lambertus et al., 2016; Pas et al., 2024; Sunness et al., 2020).

Yet, in clinical trials, accurately measuring visual functional performance is essential to assessing the true impact of treatment on real‐world visual abilities. Therefore, it is important to investigate functional biomarkers that offer the potential for a more precise evaluation of disease progression and therapeutic efficacy in STGD1 (Eriksen et al., 2026; Karuntu et al., 2024; Thompson et al., 2020).

One such functional endpoint is mesopic microperimetry (mMP), which measures retinal sensitivity at predefined retinal locations (Cedrún‐Sánchez et al., 2024; Messenio et al., 2022; Pfau, Jolly, et al., 2021; Schönbach et al., 2017; Schönbach et al., 2020). The potential of mMP as a clinical endpoint lies in its ability to detect subtle functional deficits before significant structural changes appear, making it a valuable tool for monitoring disease progression and evaluating therapeutic efficacy (Chang‐Wolf et al., 2026; Cinque et al., 2026; Csaky et al., 2019; Madheswaran et al., 2022; Yang & Dunbar, 2021). In addition to measuring retinal sensitivity, mMP tracks fixation and evaluates fixation stability, which is particularly important due to the high prevalence of fixation instability in STGD1 patients (Cicinelli et al., 2019; Midena & Pilotto, 2017; Yang & Dunbar, 2021).

To determine treatment effects using mMP as a primary endpoint, the U.S. Food and Drug Administration (FDA) has established a criterion for clinical significance, based on two expert opinions shared at a 2009 glaucoma clinical trial symposium (Weinreb & Kaufman, 2009). According to this criterion, a treatment effect is considered significant if there is a ≥7 dB change in at least five predefined, separate loci. Recently, three gene therapy clinical trials for *RPGR*‐associated X‐linked retinitis pigmentosa (XLRP) adopted this FDA criterion for evaluating treatment effects (Lam et al., 2024; Von Krusenstiern et al., 2023; Yang et al., 2024).

However, before mMP can be reliably used as a primary endpoint, it must undergo thorough assessment to ensure there are no critical flaws that could undermine the validity of clinical trial results.

The reliability of mMP as a clinical trial endpoint depends on its test–retest variability (Karuntu et al., 2025; Kong et al., 2022; Welker et al., 2018). If variability is too high, it may obscure treatment effects, making it difficult to determine whether observed changes in retinal sensitivity reflect true therapeutic benefits or measurement inconsistencies (Karuntu et al., 2025; Schönbach et al., 2020).

High variability is not only a challenge in clinical trials (Pfau et al., 2024; Yang & Dunbar, 2021) but, in clinical practice, mMP is also used as a predictor of disease progression, particularly in inherited retinal diseases (IRDs) (Yang & Dunbar, 2021).

When variability is well understood, it becomes easier to determine whether a patient is truly experiencing disease progression (Pfau et al., 2024).

This study prospectively assesses the test–retest variability of mMP in STGD1 patients and analyses factors that may impact this variability. Building on these insights, we explore the potential of mMP as a primary endpoint for evaluating novel therapeutic measures in future interventional clinical trials.

## 2 METHODS

This study used data from the STArgardt Remofuscin Treatment Trial (STARTT) (Dhooge et al., 2021). In brief, STARTT was a phase II, prospective, multicentre, randomised controlled trial that evaluated the safety and efficacy of oral soraprazan (Remofuscin) in STGD1 patients and was completed in 2022. Soraprazan is a tetrahydropyridoether small molecule that promotes degradation of lipofuscin within retinal pigment epithelium (RPE) cells by generating non‐cytotoxic reactive oxygen species (ROS), thereby reducing lipofuscin accumulation and protecting against retinal degeneration in preclinical models (Fang et al., 2022; Oh et al., 2022). For STARTT, 87 patients were recruited from six clinical study sites across Europe, including Radboud University Medical Centre Nijmegen and Leiden University Medical Centre in the Netherlands; University Eye Hospital Tübingen and the Department of Ophthalmology at the University of Bonn in Germany; the University of Southampton in the United Kingdom; and Ospedale San Raffaele in Milan, Italy.

STARTT was conducted according to the principles of the Declaration of Helsinki (Version 2013) and in accordance with the International Conference on Harmonisation (ICH) guidelines for Good Clinical Practice (GCP). The study protocol was approved by the independent ethics committees of all participating centres, including:

- CCMO, project number NL68179.091.18;
- Commissie Mensgebonden Onderzoek Arnhem‐Nijmegen, project number 2018‐4988;
- BfArM, project number 4043121;
- Ethik‐Kommission an der Medizinischen Fakultät der Eberhard‐Karls‐Universität and am Universitätsklinikum Tübingen, project number 843/2018AMG1;
- MHRA, project number 50612/001/001‐0001;
- North‐West Haydock Research Ethics Committee, project number 18/NW/0845;
- AIFA, project number 2018‐001496‐20;
- Comitato Etico Ospedale San Raffaele Milano, project number 2018‐001496‐20.

All participants provided written informed consent prior to inclusion in this study (Dhooge et al., 2021).

### 2.1 Patient selection and measurements

**FIGURE 1:**
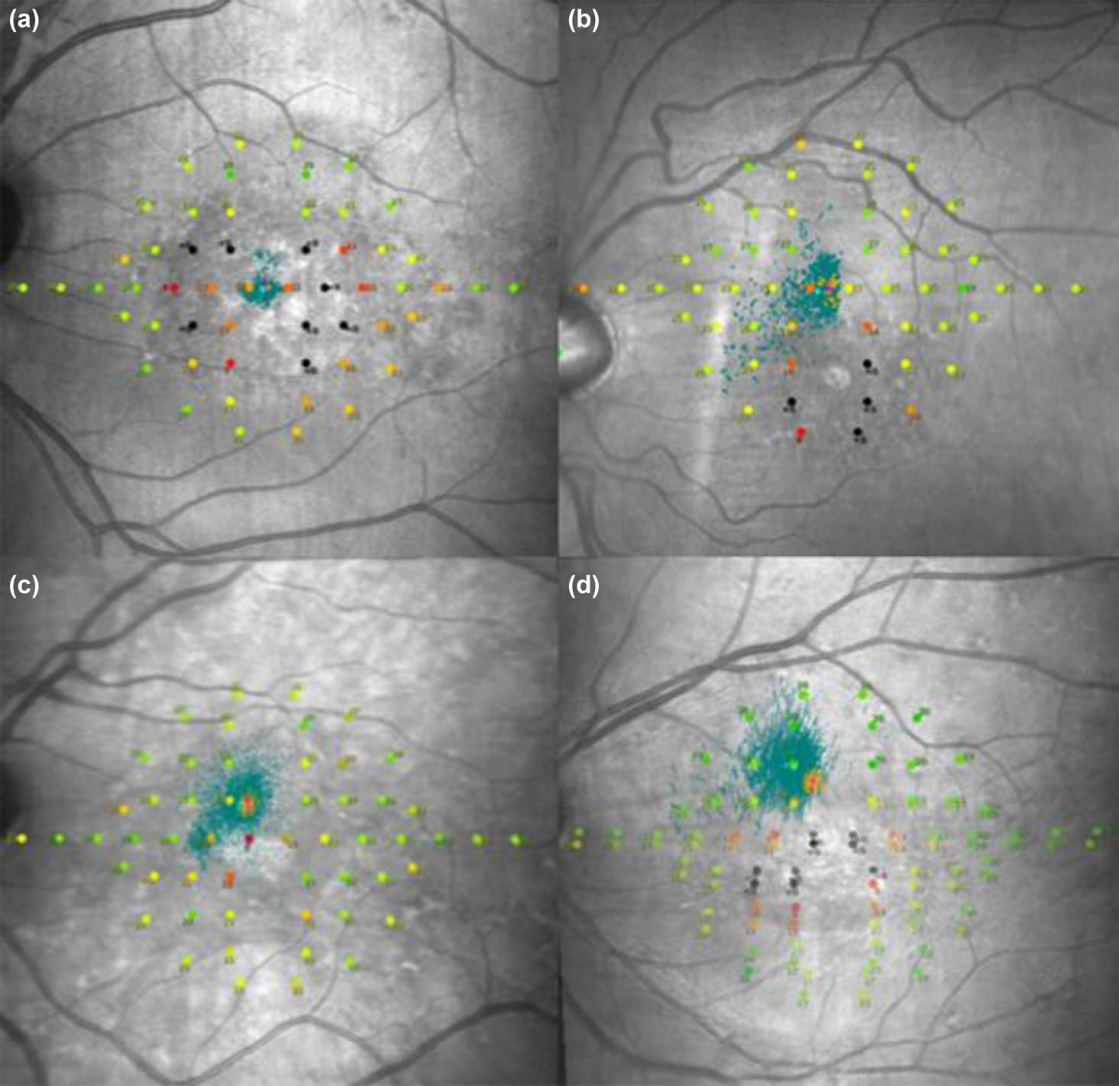
Examples of grid placement and follow‐up alignment. (a) Correct initial grid placement centred on the fovea. (b) Protocol failure due to incorrect initial grid placement. (c) Well‐aligned follow‐up with consistent grid positioning across the first and second visit. (d) Follow‐up alignment failure showing misalignment of grids between the first and second visits.

In this study, the test–retest variability was assessed between the first ‘screening’ visit (V1) and the second ‘baseline’ visit (V2) of STARTT (Dhooge et al., 2021). V1 and V2 visits of all patients took place between June 2019 and August 2020, with a median interval between visits of 21 days (IQR: 9–26 days, range: 6–63 days). Both visits occurred prior to randomisation and (sham) treatment. Exclusion criteria included absence of either V1 or V2 (incomplete data), use of an incorrect customised grid (examination failure), mMP grids not centred on the fovea within 1° of retinal eccentricity from the estimated foveal location on the infrared image (protocol failure) and misalignment of grids between V1 and V2 exceeding 1° of retinal eccentricity (follow‐up alignment failure). Protocol and follow‐up alignment failures were assessed using Fiji software (version 1.53t; National Institutes of Health, Bethesda, Maryland, United States) (Figure 1) (Bogovic et al., 2016).

### 2.2 Microperimetry assessment

**FIGURE 2:**
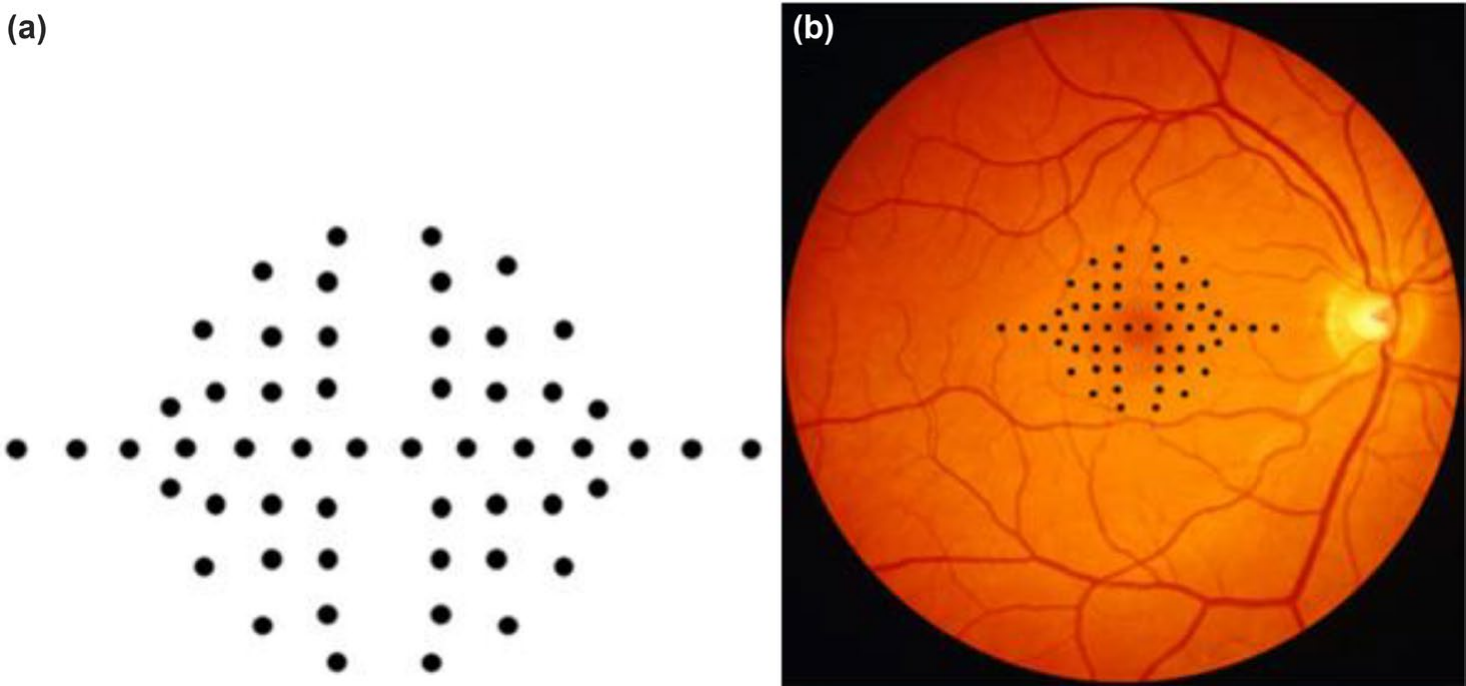
The customised grid is shown on the left (a) and is schematically overlaid on a fundus image of a healthy retina on the right (b).

Retinal sensitivity was measured with the macular integrity assessment (MAIA) microperimeter (CenterVue, Padova, Italy) after 5 minutes of dark adaptation. Retinal sensitivity at each retinal location was assessed with the 4‐2 staircase strategy, using a customised grid with 54 mesopic Goldmann III stimuli of 200 ms (Figures 1 and 2) (Convento & Barbaro, 2007, Csaky et al., 2019). Stimuli were presented on a logarithmic decibel (dB) scale, ranging from the brightest (0 dB) to the least bright (36 dB) (Charng et al., 2020).

Prior to testing study subjects, each technician underwent individual operator certification by performing tests in accordance with the predefined mMP protocol on two test eyes, one of which had retinal pathology. The test results were reviewed by the Central Reading Centre for plausibility, quality and completeness before certification was granted. For subject testing, the fellow eye was always tested before the study eye. At each study visit, testing began with a brief training session, consisting of eight stimuli, serving as a warm‐up to minimise learning effects, followed by customised grid testing with 54 stimuli (Wong et al., 2017; Wu et al., 2013). At the second visit, the MAIA follow‐up mode was used for mMP testing to ensure automated retesting of the same retinal locations identified during the first visit (Yang & Dunbar, 2021).

### 2.3 Retinal sensitivity calculations

Both *pointwise sensitivity* (*PWS*) and *mean sensitivity* (*MS*) were determined from the MAIA data output after each session. *PWS* refers to the recorded sensitivity (in dB) at each stimulus location (Chen et al., 2009; Coulibaly et al., 2024; Taylor et al., 2023). *MS* was calculated as the average sensitivity threshold across all 54 stimuli (Figure 2) (Taylor et al., 2023). Absolute scotomatous loci were defined as points with no perception of stimuli at the highest tested intensity, indicated as <0 dB in the manufacturer's data output. For calculation purposes, <0 dB values were converted to −1 dB (Charng et al., 2024).

### 2.4 Test–retest variability

To evaluate test–retest variability, Bland–Altman analyses were performed. Limits of agreement (mean difference ± 1.96 × the within‐subject standard deviation (S_W_)) were calculated and visualised against their average (Bland & Altman, 1986). Subsequently, coefficients of repeatability (CoR) were determined by multiplying the within‐subject standard deviation (S_W_) by $\sqrt{2}$ × 1.96 (Beckerman et al., 2001; Bland, 2015; Bland & Altman, 1986; Lexell & Downham, 2005). The CoR represents the threshold below which the absolute difference between V1 and V2 is expected to fall with 95% probability (Vaz et al., 2013).

For *PWS*, variability was calculated as the difference between V1 and V2 (V2 minus V1) at each locus. For *MS*, variability was determined as the difference between V1 and V2 (V2 minus V1) across the entire grid per eye.

Additionally, the CoR for each *PWS* value was assessed by calculating the CoR between *PWS* values from V1 and V2 and plotting it against the average *PWS* from both visits. The CoR and *PWS* values were also analysed at each specific locus position. Furthermore, for each locus in the grid, the percentage of participants exhibiting an absolute change of ≥7 dB was calculated, consistent with the FDA criterion for treatment effect (≥7 dB change in ≥5 predefined, separate loci) (Weinreb & Kaufman, 2009).

### 2.5 Factors influencing test–retest variability

#### 2.5.1 Learning effect

Learning effect was assessed by calculating the mean difference (bias) between V1 and V2 (V2 minus V1) across all retinal sensitivity metrics (Wong et al., 2017; Wu et al., 2013). A positive bias, indicating higher retinal sensitivity metrics in V2 compared to V1, suggested the presence of a learning effect (Wong et al., 2017, Wu et al., 2013).

#### 2.5.2 Fixation stability

To assess fixation stability and its potential influence on test–retest variability, bivariate contour ellipse area (*BCEA*) *63* and *95* (deg^2^), *P1* (%), *P2* (%) *and fixation losses* (%) were measured during each session and subsequently analysed.

*BCEA 63* and *BCEA 95* quantify the scattering of fixation points within an ellipse encompassing 63% and 95% of the points around the fixation target (Crossland et al., 2009). *P1* and *P2* represent the percentage of fixation points falling within a circle of 1° (*P1*) or 2° (*P2*) diameter around the fixation target (Fujii et al., 2002).

*P1* and *P2* were clinically stratified into *stable fixation* (*P1* ≥ 75% and *P2* ≥ 75%), *relatively unstable fixation* (*P1* < 75% and *P2* ≥ 75%) or *unstable fixation* (*P1* < 75% and *P2* < 75%) (Fujii et al., 2002).

*Fixation losses* (%) were measured using the *Heijl–Krakau* method by detecting false‐positive responses to 10 dB stimuli at the optic disc (Heijl & Krakau, 1975).

#### 2.5.3 Other factors

Best‐corrected visual acuity (BCVA) was assessed using an early treatment diabetic retinopathy study (ETDRS) chart at 4 m distance. The ETDRS score was converted into LogMAR for analysis. The average from V1 and V2 was used for each eye.

*Average reaction time* (ms) was determined by calculating the mean of the average reaction time per stimulus from V1 and V2 for each eye. *Exam duration* (s) was determined by calculating the average exam duration from V1 and V2 for each eye.

### 2.6 Statistical analysis

Statistical analyses were performed using IBM SPSS Statistics (version 29; IBM Corp., Armonk, New York, United States). All variables were assessed for normality using histograms and Shapiro–Wilk analyses. Parametric or non‐parametric analyses were applied as appropriate. Parametric data are presented as mean and ± standard deviation (SD) and non‐parametric data as median and interquartile range (IQR). For all statistical analyses, significance was defined as *p* < 0.05. Bonferroni correction was applied when appropriate to control for Type I error associated with multiple comparisons.

## 3 RESULTS

### 3.1 Participant demographics

**FIGURE 3:**
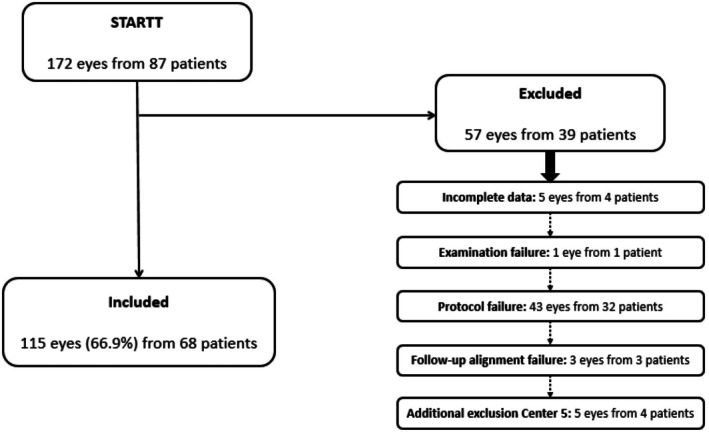
Overview of patient inclusion and exclusion.

A total of 115 eyes from 68 subjects were included in the study (Figure 3). Unfortunately, a large proportion of the data (33.14%) had to be excluded, primarily due to protocol failure, which was defined as the grid not being centred on the fovea (i.e. more than 1° away from the estimated foveal location on the infrared image), which affected 43 eyes from 32 patients. Additional exclusions were due to incomplete data (i.e. missing V1 or V2 data; 5 eyes from 4 patients), examination failure with an aberrant grid (1 eye) and misalignment of grids between V1 and V2 (3 eyes from 3 patients).

Because the majority of eyes from one centre were excluded due to protocol failure, the remaining initially included eyes from that centre (5 eyes from 4 patients) were also excluded to minimise potential bias.

A comprehensive overview of the characteristics of this study cohort is provided in Table 1.

**TABLE 1:** Study cohort characteristics.

Characteristics	Included patients (*n* = 68)^a^	Excluded patients (*n* = 49)^a^	*p*‐value
Gender (female/male, %)	39 (57.4)/29 (42.6)	22 (56.4)/17 (43.6)	N/A
Age at informed consent (years)	36.15 ± 11.38	36.38 years ±12.17	N/A
Age at diagnosis (years)	28.19 years ±8.27	27.33 years ±8.26	N/A
Disease duration (IQR)	5 years (2.25–10)	6 years (3–11)	N/A
Ethnicity (Caucasian/Asian/Hispanic)	97.1%/1.5%/1.5%	92.3%/5.1%/2.6%	N/A
BCVA (IQR)	0.34 LogMAR (0.20–0.70)	0.62 LogMAR (0.32–0.70)	*p* = 0.004
*BCEA 63* (IQR)	2.29 deg^2^ (0.36–5.40)	5.74 deg^2^ (2.79–10.87)	*p* < 0.001
*BCEA 95* (IQR)	6.87 deg^2^ (1.07–16.22)	17.21 deg^2^ (8.35–32.58)	*p* < 0.001

*Note*: Data presented as mean ± standard deviation (SD), interquartile range (IQR), or *n* (%). BCVA refers to best‐corrected visual acuity, and *BCEA* represents the bivariate contour ellipse area. The Mann–Whitney *U*‐test was used to compare BCVA and *BCEA* values between included and excluded patients.

^a^ Patients may appear in both groups, as inclusion or exclusion was determined per eye.

### 3.2 Test–retest variability

#### 3.2.1 Mean sensitivity (MS) and pointwise sensitivity (PWS)

**FIGURE 4:**
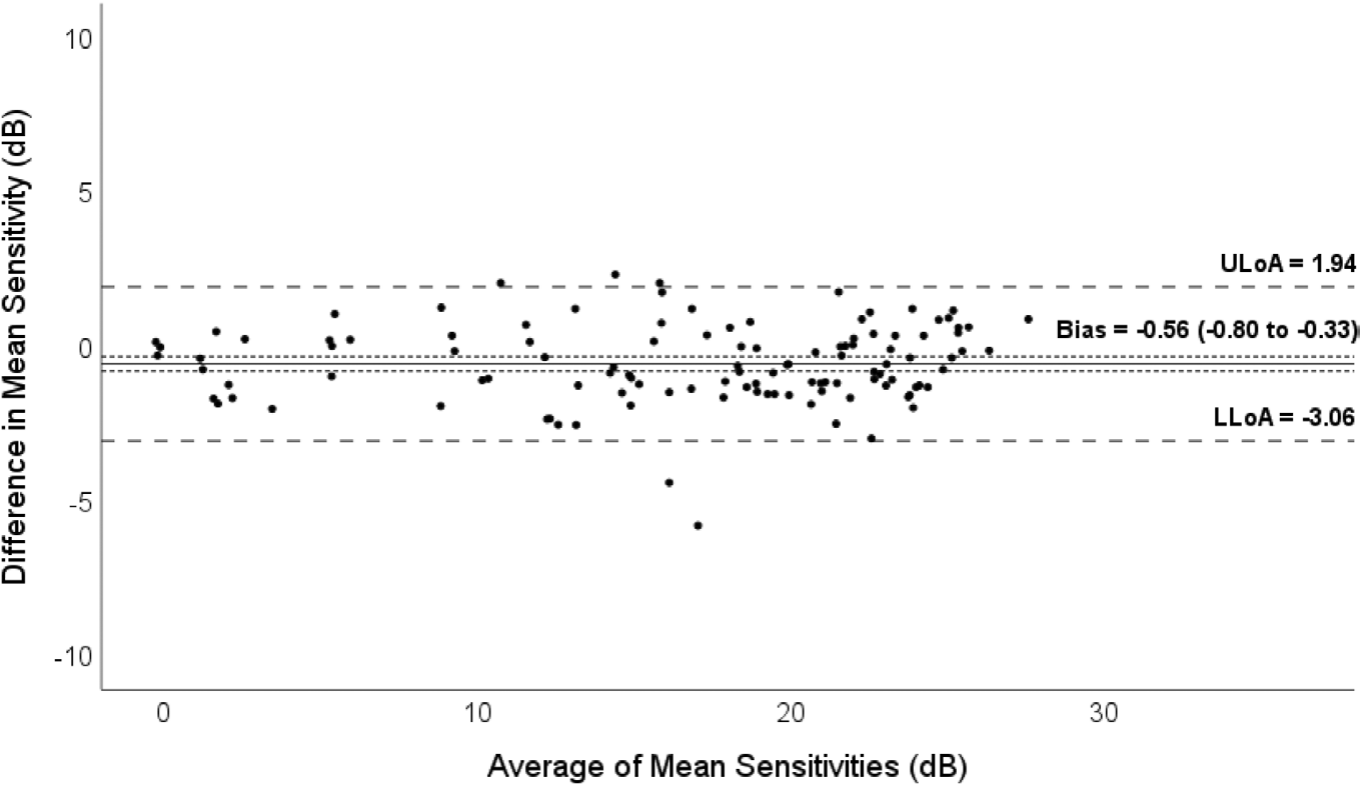
Bland–Altman plot showing the average of *MS* from V1 and V2 on the x‐axis, plotted against the difference between V1 and V2 (V2 minus V1) on the y‐axis. ULoA: Upper limit of agreement, bias: Mean difference between V1 and V2 (V2 minus V1), LLoA: Lower limit of agreement.

**FIGURE 5:**
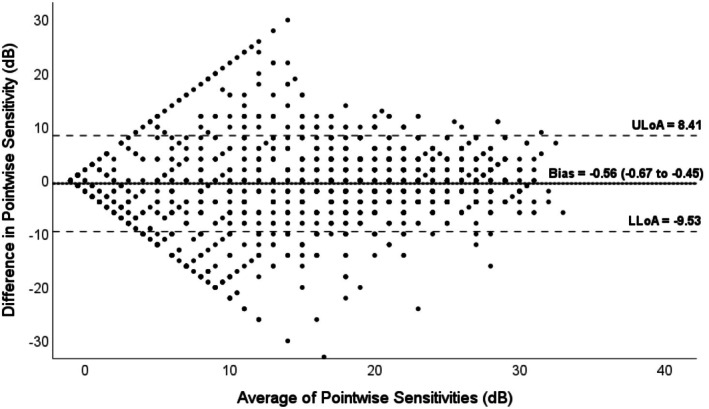
Bland–Altman plot of *PWS* showing the average of *PWS* from V1 and V2 on the x‐axis, plotted against the difference between V1 and V2 (V2 minus V1) on the y‐axis. ULoA: Upper limit of agreement, bias (95% CI): Mean difference between V1 and V2 (V2 minus V1), LLoA: Lower limit of agreement.

**FIGURE 6:**
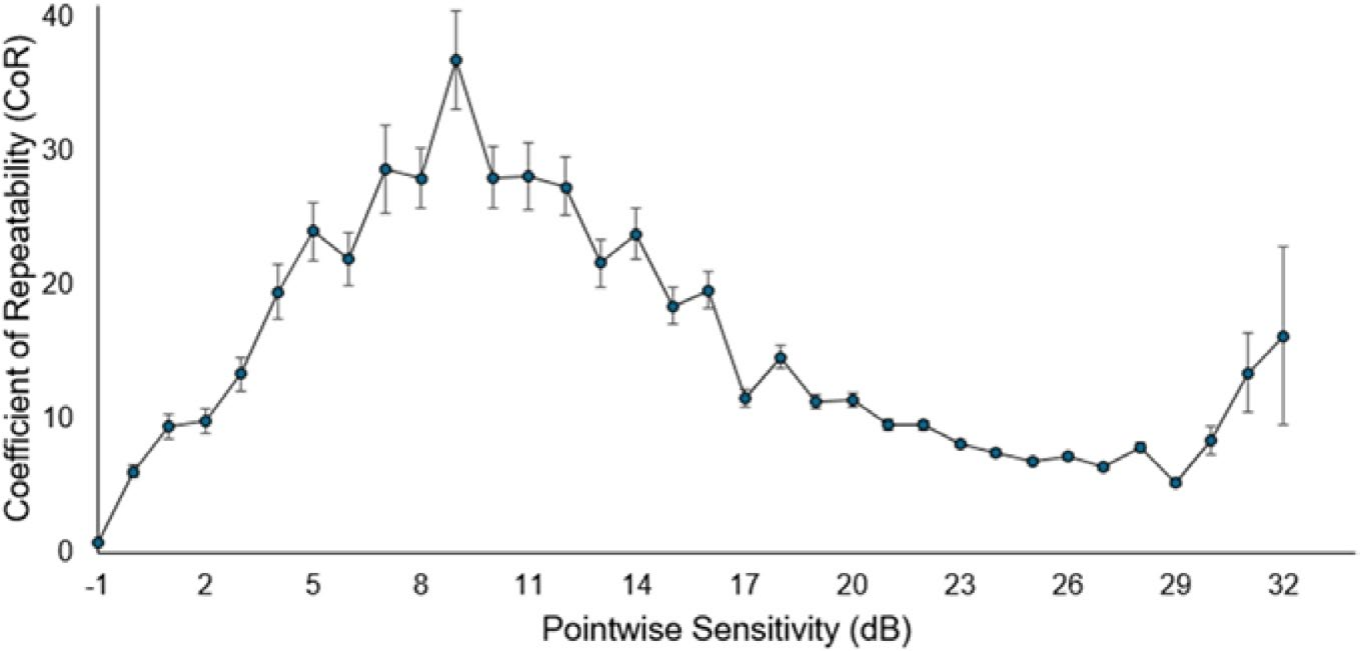
Retinal sensitivity slope showing the coefficient of repeatability (CoR) per *pointwise sensitivity* (*PWS*) value (dB). The graph illustrates lower test–retest variability at lower *PWS* values (−1 dB to 3 dB) and higher *PWS* values (16–32 dB). Most test–retest variability occurred at *PWS* values between 4 dB and 16 dB, peaking at 9 dB (CoR: 36.86 dB).

Table 2 summarises the *mean sensitivity* (*MS*) and *pointwise sensitivity* (*PWS*). *MS* had the lowest test–retest variability (CoR: 3.53 dB, 95% CI: 3.07–3.99) and an average retinal sensitivity value of 16.67 dB ± 7.39 dB (Figure 4 and Table 2). In contrast, *PWS* exhibited the highest test–retest variability (CoR: 12.69 dB, 95% CI: 12.47–12.91) (Figure 5 and Table 2). Most test–retest variability occurred in *PWS* values between 4 dB and 16 dB, peaking at 9 dB (CoR: 36.86 dB, 95% CI: 33.21–40.51) (Figure 6).

**TABLE 2:** Summary of *MS* and *PWS*.

Metric	Mean ± SD (dB)^a^	CoR (dB)	95% Limits of Agreement (dB)	Bias (dB)
Mean sensitivity (MS)	16.67 ± 7.39	3.53 (3.07 to 3.99)	1.94 to −3.06	−0.56 (−0.79 to −0.33)
Pointwise sensitivity (PWS)	16.68 ± 10.44	12.69 (12.47 to 12.91)	8.41 to −9.53	−0.56 (−0.67 to −0.45)

*Note*: Data are presented with the 95% confidence interval shown in brackets.

^a^ Mean of the first and second visits. CoR: Coefficient of Repeatability, dB: decibel.

*PWS* had a mean retinal sensitivity value of 16.68 dB (±SD: 10.44), with values increasing from the central to peripheral regions, ranging from 6.36 dB to 22.90 dB (Table 2 and Appendix S3).

The CoR per locus for all participants exceeded the FDA threshold of ≥7 dB across all loci (Appendix S1). Furthermore, a substantial proportion of participants demonstrated absolute test–retest variability of ≥7 dB at each anatomical locus, with proportions reaching up to 25.86% at the central grid and progressively decreasing towards the periphery (Appendix S2).

### 3.3 Factors influencing test–retest variability

#### 3.3.1 Fixation stability

A statistically significant weak positive correlation was observed between *fixation losses* and test–retest variability (Spearman's *ρ* = 0.284, *p* = 0.003) (Appendix S4). In contrast, *BCEA 63* and *BCEA 95* were not significantly correlated with test–retest variability (*BCEA 63*: Spearman's *ρ* = 0.162, *p* = 0.095; *BCEA 95*: Spearman's *ρ* = 0.164, *p* = 0.092). Furthermore, no significant difference in test–retest variability was found among the *stable*, *relatively unstable* and *unstable* fixation groups (Kruskal–Wallis test: *p* = 0.292).

#### 3.3.2 Other factors

A statistically significant strong positive correlation was observed between *exam duration* and test–retest variability (Spearman's *ρ* = 0.609, *p* < 0.001). *Exam duration* was also significantly positively correlated with *fixation losses*, although the correlation was weak (Spearman's *ρ* = 0.356, *p* < 0.001) (Appendix S4). In contrast, BCVA and *average reaction time* were not significantly correlated with test–retest variability.

## 4 DISCUSSION

Currently, mMP emerges as a potential endpoint for trials involving IRDs such as STGD1; however, its clinical utility is increasingly scrutinised (Schönbach et al., 2020). This study provides key insights into the reliability of mMP and its potential for measuring treatment effects and monitoring disease progression in STGD1. We evaluated several factors that may influence the reliability of mMP and found that test–retest variability is strongly dependent on the specific outcome metric used.

The highest overall test–retest variability was observed in *pointwise sensitivity* (*PWS*) with a CoR of 12.69 dB (95% CI: 12.47–12.91). This aligns closely with the previously reported CoR of 12.15 dB in STGD1 also using the MAIA microperimeter, as described by Pfau et al. (Pfau, Holz, & Müller, 2021). In contrast, this value is considerably higher than those reported in other retinal diseases, such as intermediate age‐related macular degeneration (iAMD) (CoR: 4.4 dB) and geographic atrophy (GA) due to AMD (CoR: 6.57 dB), also measured with the MAIA system (Coulibaly et al., 2025; Welker et al., 2018). The high test–retest variability could pose a challenge, as reliably detecting changes in specific retinal regions that deteriorate over the course of a trial is crucial for assessing disease progression and treatment efficacy.

*Mean sensitivity* (*MS*) demonstrated the lowest test–retest variability, with a CoR of 3.53 dB (95% CI: 3.07–3.99). Another recent study by Pfau et al. described a slightly lower CoR of approximately 2 dB in *MS* obtained from a large dataset (531 eyes of 432 healthy adults) from five independent study groups using several types of grids (Pfau et al., 2024). Other studies in patients with different macular diseases, using a different mMP system (Nidek MP‐1), reported CoRs ranging from 1.65 dB to 1.81 dB (Chen et al., 2009; Georgiou et al., 2020). Given its proven lower variability, this global metric might be a useful tool for tracking overall functional changes across the central retina.

Yet, *MS* has important limitations. Localised functional changes may be obscured by absolute scotomas or points near the measurement floor (≈0 dB), while the inclusion of normal test points can attenuate treatment effects on diseased areas, thereby reducing the likelihood of demonstrating efficacy in clinical trials (Barkana et al., 2021; Charng et al., 2024; Josan et al., 2021; Taylor et al., 2023). Additionally, *MS* does not account for the spatial distribution of retinal sensitivity values across grid points, which is particularly limiting when using customised grids with unequal spacing (Josan et al., 2021).

Volume sensitivity may offer an alternative by integrating both retinal sensitivity values and their spatial distribution, thereby avoiding the skewing effect of absolute scotomas observed in *MS* (Josan et al., 2021; Karuntu et al., 2025). This approach could increase sensitivity to localised functional changes over time (Josan et al., 2021). However, the application and interpretation of volume sensitivity can be more complex (Josan et al., 2021; Yang & Dunbar, 2021). Similar to *MS*, the need to assess a large number of grid points may elevate the testing burden and, consequently, test–retest variability.

These findings suggest that measurement strategies specifically designed for STGD1 could be beneficial. An alternative approach for evaluating treatment effect with mMP could involve combining multiple grid points into a disease‐specific pattern. In the case of STGD1, this might involve using a ring‐shaped array around the fovea (parafoveal ring). Such a configuration may help reduce the CoR by requiring fewer grid points, thereby limiting test duration while specifically targeting regions of disease progression. Further research is needed to validate this strategy and determine its potential to more accurately capture treatment effects in STGD1.

However, when evaluating such strategies, it is essential to consider how they align with current regulatory benchmarks. The FDA threshold for clinical significance in clinical trials (≥7 dB change in ≥5 predefined, separate loci) may have limited applicability to STGD1 (Lam et al., 2024; Von Krusenstiern et al., 2023; Weinreb & Kaufman, 2009; Yang et al., 2024). In our study, the *PWS* CoR was 12.69 dB (95% CI: 12.47–12.91). Furthermore, CoRs at all anatomical positions exceeded the ≥7 dB threshold. Notably, within the central grid, up to 25.86% of participants exhibited absolute test–retest variability of ≥7 dB per locus.

### 4.1 Influencing factors

We demonstrated the presence of a floor and ceiling effect in this study cohort, where test–retest variability decreases as retinal sensitivity values approach the minimum (−1 dB to 3 dB) or relative maximum (16–32 dB) levels of mMP. Highest test–retest variability occurred at *PWS* values between 4 dB and 16 dB, peaking at 9 dB (CoR: 36.86 dB, 95% CI: 33.21–40.51). These findings align with those reported by Pfau et al., who observed the highest variability between the MS >4–6 dB and >16–18 dB groups, peaking at >10–12 dB (CoR: approx. 26 dB) and decreasing towards both the minimum and maximum *MS* values (Pfau, Holz, & Müller, 2021). While Pfau et al. used *MS*, which averages differences in *PWS* across the assessed grid, their results, combined with our observations, further support the presence of both floor and ceiling effects in test–retest variability with mMP in STGD1 patients (Pfau, Holz, & Müller, 2021).

Building on this, we also examined additional factors that may influence variability within our cohort. Notably, *exam duration* showed a strong positive correlation with test–retest variability (Spearman's *ρ* = 0.609, *p* < 0.001), possibly due to increased fatigue as testing time lengthens. This fatigue may lead to more *fixation losses*, as prolonged testing demands sustained concentration. This could explain the weak positive correlation between *fixation losses* and test–retest variability (Spearman's *ρ* = 0.284, *p* = 0.003) as well as the weak positive correlation between *exam duration* and *fixation losses* (Spearman's *ρ* = 0.356, *p* < 0.001).

A possible solution to reduce test–retest variability is to shorten the duration of the mMP test by reducing the number of stimuli in the tested grid. We recommend an exam duration of ≤450 seconds, as our study showed a substantial increase in test–retest variability for mMP tests exceeding this duration. Furthermore, no learning effect was observed in this study, as no retinal sensitivity metric demonstrated a significant positive mean difference (V2 minus V1) in retinal sensitivity between V1 and V2.

Interestingly, other fixation metrics such as *BCEA 63*, *BCEA 95*, *P1* and *P2* did not show a correlation with test–retest variability in this study. This contrasts with the significant correlation between *BCEA 95* and test–retest variability reported by Josan et al. (Josan et al., 2023).

### 4.2 Limitations

Unfortunately, a large proportion of test results could not be used, despite assessments being conducted within the framework of a clinical trial. Three quarters of the excluded patients were removed due to the fovea not being centred on the grid. Notably, the excluded group exhibited significantly greater fixation instability, with mean *BCEA 63* and *BCEA 95* values of 5.74 deg^2^ and 16.60 deg^2^, respectively, compared to 2.29 deg^2^ and 6.87 deg^2^ in the included group.

This suggests that the manual step of moving the exact centre of the grid from the fixation point to the anatomical fovea was omitted or performed incorrectly. This issue could have been avoided with improved operator training in mMP acquisition. Furthermore, the quality control process could have benefited from direct assessment of images by the Central Reading Centre, enabling earlier identification and correction of alignment errors.

As a mitigation strategy to minimise selection bias in future studies, we recommend comprehensive operator training in mMP usage, with an emphasis on accurately centring the grid on the fovea, especially in patients with eccentric fixation.

Integrating optical coherence tomography (OCT) images with precise foveal demarcation into the mMP system may also assist operators in accurately determining foveal location, thereby improving alignment and measurement reliability. In addition, Central Reading Centre review of the initial mMP measurement could facilitate early detection of insufficient data, allowing for reassessment before the trial progresses.

Beyond acquisition quality, another important consideration is the long‐term consistency of mMP measurements. In the current study, we assessed test–retest variability between only two tests conducted within a relatively short interval (median: 21 days, IQR: 9–26 days). Since clinical trials typically span several years and require visits at intervals of a few months, future studies should investigate test–retest variability over multiple visits and extended periods.

## 5 CONCLUSIONS

Our findings suggest that, given its high test–retest variability, the use of *PWS* should be carefully considered for clinical application and future clinical trials. Although *MS* demonstrates lower variability, it may fail to capture localised functional changes. Additionally, alternative measures of treatment effect may be worth exploring, as the current FDA criterion of a ≥ 7 dB change in at least five predefined, separate loci may not adequately reflect the variability observed in STGD1 patients. A novel mMP strategy for STGD1, such as employing a parafoveal ring that combines and averages multiple grid points, may be advantageous but requires further validation. To enhance consistency and reduce variability, we recommend limiting mMP test duration to ≤450 seconds and ensuring comprehensive operator training to support proper grid alignment on the fovea. In conclusion, the reliability of mMP as a clinical endpoint strongly depends on the outcome metric (*PWS* or *MS*) used, with factors such as test duration, fixation losses, retinal sensitivity values and anatomical location influencing variability.

## AUTHOR CONTRIBUTIONS

Conceptualisation: SHK, JAAHP and CBH. Data curation: SHK, JAAHP, PPAD and CBH. Formal analysis: SHK and JAAHP. Funding acquisition: CBH. Investigation: PPAD, SSV, MBP, PH, FGH, AJL, KS, THWS, CJFB and CBH. Methodology: SHK, JAAHP, PPAD and CBH. Project administration: SHK, JAAHP and CBH. Software: SHK and JAAHP. Supervision: CBH. Validation: SHK and JAAHP. Visualisation: SHK, JAAHP, PPAD and CBH. Writing—original draft: SHK, JAAHP, PPAD and CBH. Writing—review and editing: SHK, JAAHP, PPAD, SSV, MBP, PH, FGH, AJL, KS, THWS, CJFB and CBH.

## FUNDING INFORMATION

This study was conducted as part of STARTT and was funded by the European Union's Horizon 2020 Research and Innovation Programme under Grant Agreement No. 779317.

## Supporting information


Appendix S1.

## Data Availability

The data that support the findings of this study are available from the corresponding author upon reasonable request.
