# Supplementary material for: Mesopic microperimetry in Stargardt disease: Application and reliability
Source: Acta Ophthalmol. 2026 Jan 29;104(5):e544–54. doi: 10.1111/aos.70072 (PMC13353732; doi:10.1111/aos.70072)
Supplement: Supplementary file 1 — Appendix S1. [file AOS-104-e544-s001.docx]

**A****ppendices**

**APPENDIX 1.** CoR for each specific locus position


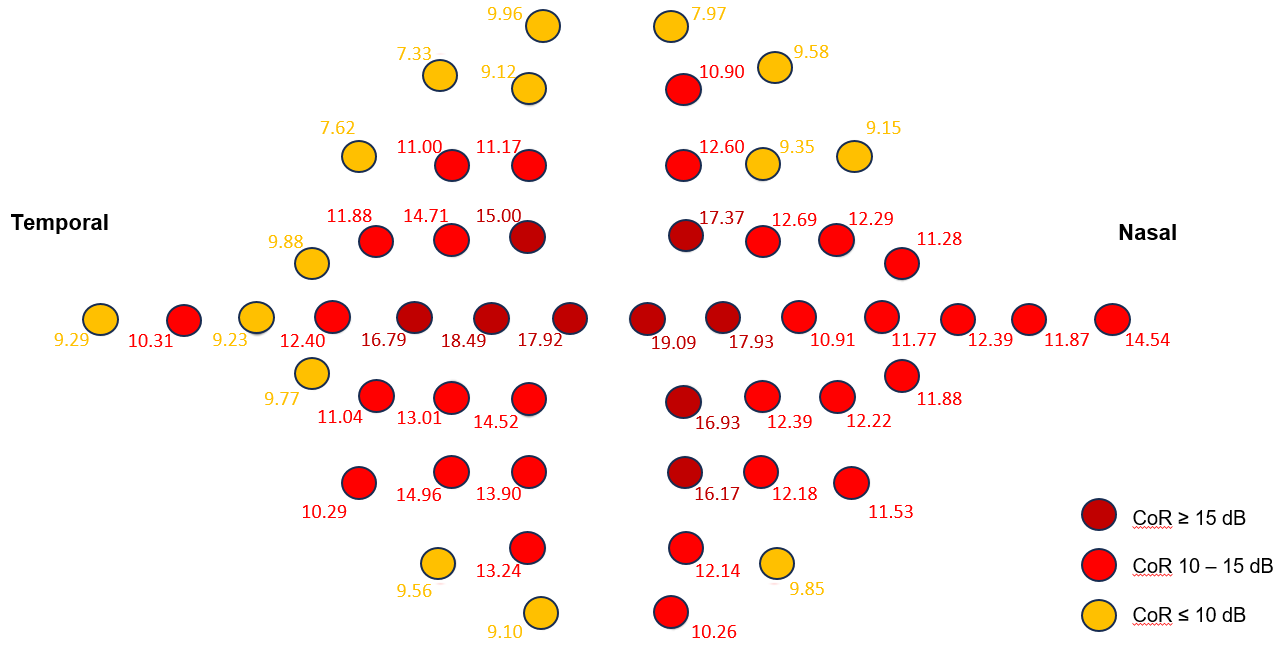


**APPENDIX 1.** **Schematic view of the testing grid illustrating the coefficient of repeatability (CoR) per locus. The CoR values range from the lowest (7.33 dB) in the periphery to the highest (19.09 dB) in the centre of the grid. The CoR per locus for all participants exceeded the FDA threshold (≥7 dB difference) across all loci. The high CoR at the most nasal location is attributed to overlap with the optic disc margin in multiple participants.**

**APPENDIX 2.** Percentage of participants with ≥7 dB difference per locus


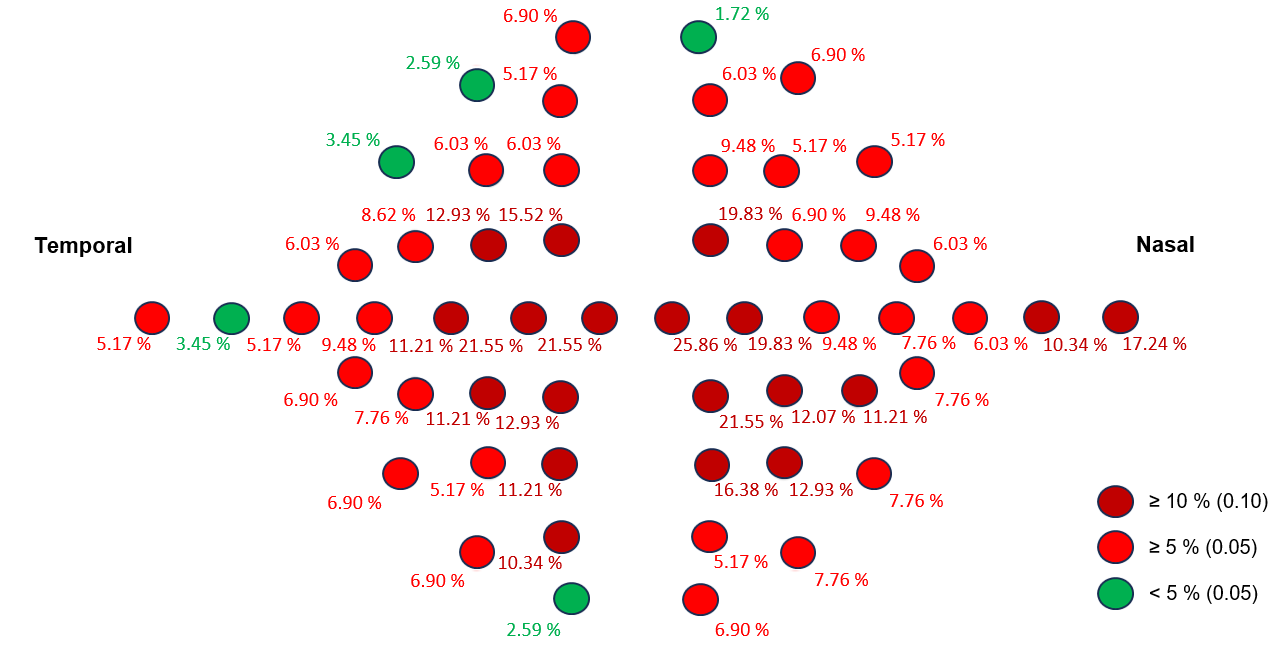


**APPENDIX 2.** **Schematic view of the testing grid illustrating the percentage of participants with an absolute variability of** **≥7 dB between V1 and V2 per locus. The high percentage at the most nasal location is attributed to overlap with the optic disc margin in multiple participants.**

**APPENDIX 3. *PWS* per locus**


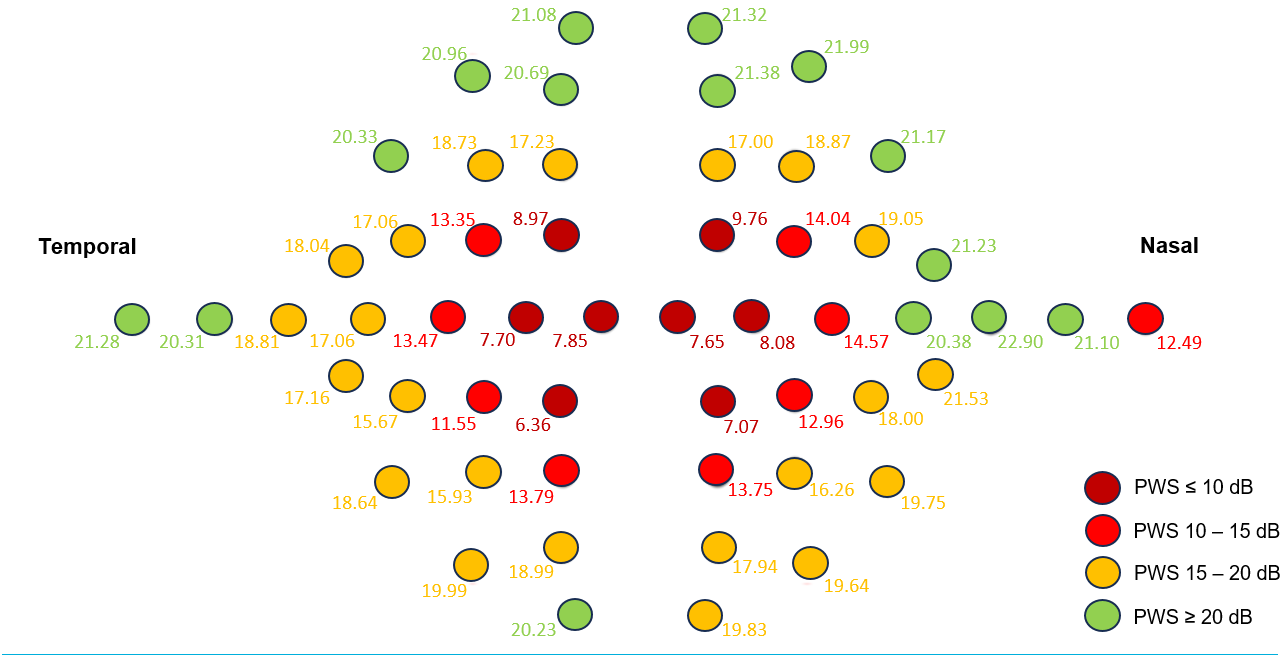


**APPENDIX 3. Schematic view of the testing grid illustrating the *pointwise sensitivity* (*PWS*) per locus. *PWS* increases from the central to peripheral regions, ranging from 6.36 dB to 22.90 dB. Low *PWS* at the most nasal location is attributed to overlap with the optic disc margin in multiple participants.**

**APPENDIX 4. Scatterplots *exam duration* and *fixation losses***

A


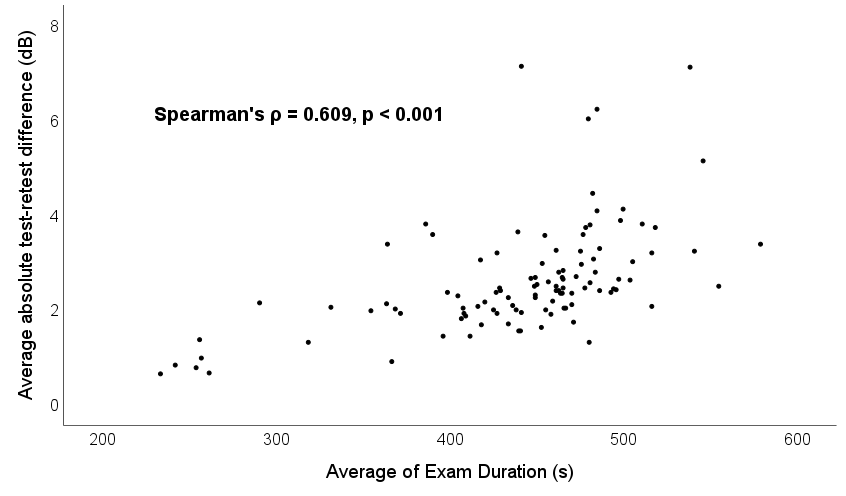


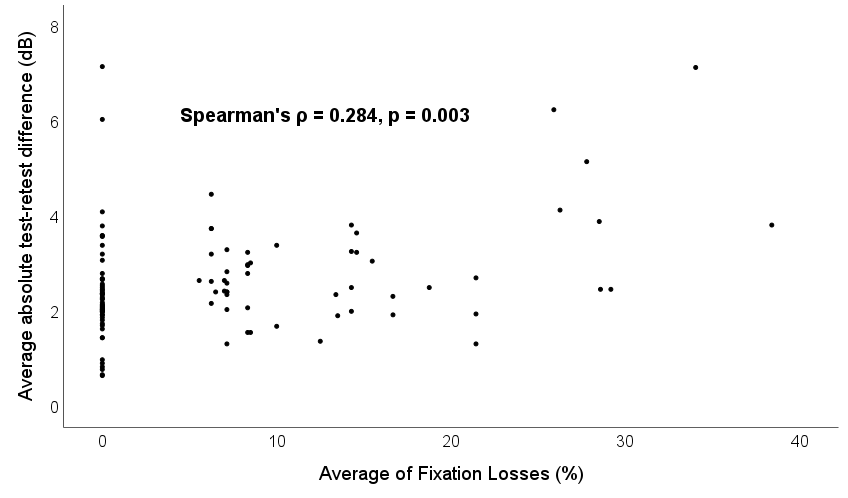


B


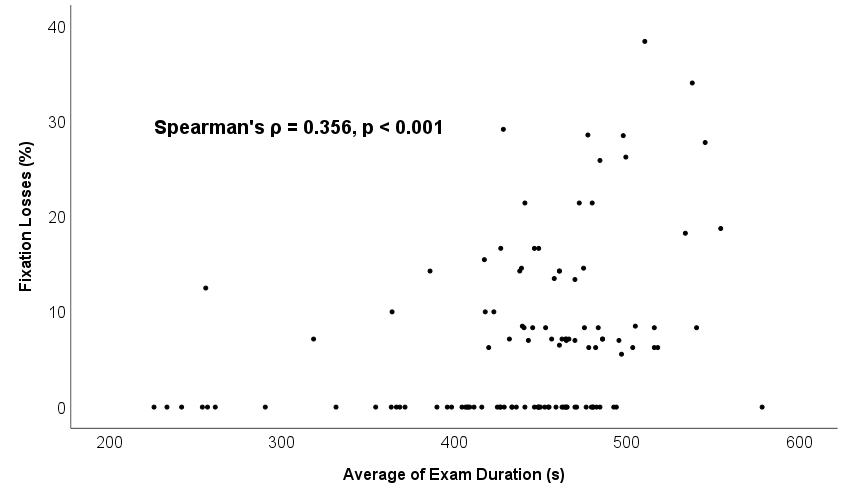


C

**APPENDIX 4.** The top two scatterplots show the average *exam duration* (A) and average *fixation losses* (B) on the x-axis, plotted against the absolute test-retest difference between V1 and V2 (V2 minus V1). The bottom scatterplot shows the average *exam duration* (C) on the x-axis, plotted against *fixation losses* between V1 and V2 (V2 minus V1).
